# Progress Toward Identifying Exact Proxies for Predicting Response to Immunotherapies

**DOI:** 10.3389/fcell.2020.00155

**Published:** 2020-03-17

**Authors:** Aleksandra Filipovic, George Miller, Joseph Bolen

**Affiliations:** ^1^PureTech *Health* PLC, Boston, MA, United States; ^2^New York University School of Medicine, New York, NY, United States

**Keywords:** immunotherapy, checkpoint inhibitors, biomarkers, cancer, PD-1, PDL-1, CTLA-4

## Abstract

Clinical value and utility of checkpoint inhibitors, a drug class targeting adaptive immune suppression pathways (PD-1, PDL-1, and CTLA-4), is growing rapidly and maintains status of a landmark achievement in oncology. Their efficacy has transformed life expectancy in multiple deadly cancer types (melanoma, lung cancer, renal/urothelial carcinoma, certain colorectal cancers, lymphomas, etc.). Despite significant clinical development efforts, therapeutic indication of approved checkpoint inhibitors are not as wide as the oncology community and patients would like them to be, potentially bringing into question their universal efficacy across tumor histologies. With the main goal of expanding immunotherapy applications, identifying of biomarkers to accurately predict therapeutic response and treatment related side-effects are a paramount need in the field. Specificities surrounding checkpoint inhibitors in clinic, such as unexpected tumor response patterns (pseudo- and hyper-progression), late responders, as well as specific immune mediated toxicities, complicate the management of patients. They stem from the complexities and dynamics of the tumor/host immune interactions, as well as baseline tumor biology. Search for clinically effective biomarkers therefore calls for a holistic approach, rather than implementation of a single analyte. The goal is to achieve dynamic and comprehensive acquisition, analyses and interpretation of immunological and biologic information about the tumor and the immune system, and to compute these parameters into an actionable, maximally predictive value at the individual patient level. Limitation delaying swift incorporation of validated immuno-oncology biomarkers span from standardized biospecimens acquisition and processing, selection of proficient biomarker discovery and validation methods, to establishing multidisciplinary consortiums and data sharing platforms. Multi-disciplinary efforts have already yielded some approved (PDL-1 and MSI-status) and other advanced tests (TMB, neoantigen pattern, and TIL infiltration rate). Importantly, clinical trial taskforces now recognize the imperative of the biomarker-driven trial design and execution, to enable translating biomarker discoveries into the clinical setting. This will ensure we utilize the “conspiracy” between the peripheral and intra-tumoral dynamic markers in shaping responses to checkpoint blockade, for the ultimate patient benefit.

## Introduction

The immune system holds remarkable potential to recognize and destroy cancer cells, but the complex network governing tumor immune escape is an obstacle to broadly effective immune modulation (Martinez-Bosch et al., 2018). Cellular signals traveling through PD-1/PDL-1 and CTLA-4 axes work by smothering T cell activation, which in turn, prohibits tumor-directed adaptive immune response. In light of unprecedented efficacy observed in multiple, historically difficult to treat tumor types using PD-1, PDL-1 and CTLA-4, inhibitors (CPIs), we now benefit from seven U.S. FDA approved drugs across sixteen indications between years 2011 and 2019 (Xiao et al., 2020). These agents are now integrated as standard of care in addition to and in conjunction with surgery, chemotherapy, targeted molecules and radiotherapy (George et al., 2019). Despite significant clinical development efforts, therapeutic indication of approved checkpoint inhibitors are still limited, potentially bringing into question their universal efficacy across tumor histologies. Recent broad meta-analyses revealed some expected and other surprising facts. As predicted, the adoption rate of checkpoint inhibitors steeply increased with patient eligibility climbing from 1.54 to 43.63% in the space of nine years (2011–2018) (Haslam and Prasad, 2019). However, we may have expected that response rates would follow an equally impressive trend. While percent of patient responding, registered at ∼13% in 2018, does represent a considerable improvement from the negligible <1% of cancer bearing individuals having benefit from CPIs in 2011, expectation of the oncology community and patients are aimed at achieving a much better result. Treatment with checkpoint inhibitors has shifted the therapeutic paradigm in oncology, as we witnessed distinct response patterns and duration of responses elicited by unleashing immune mediated cancer attack. Namely, in nineteen studies involving >11,000 patients treated across 42 treatment arms, the number of patients who experienced a durable response to checkpoint inhibitors was 2.3 times, compared to any of the agents used as standard of care in the control arms (11%). Furthermore, anti-PD-1/PDL-1 agents tend to yield longer lasting responses over anti-CTLA-4 agents (Siu et al., 2017; Catenacci et al., 2019). Taking this into consideration, experience with the first generation of checkpoint inhibitors has awoken us to the fact that more sophisticated, immune relevant markers are needed for efficient patient assignment to ensure more patients experience these benefits.

A patient’s peripheral and intra-tumoral immunity form an interface between the cancer and the host, and tumor-host immune interactions play an indispensable role in carcinogenesis, metastasis and determination of the response to treatment. Tumor tissue immune landscape and the peripheral blood immune cells form the so-called “immunome,” the analyses of which need to encompass a broad range of test strategies to capture: (i) target biology; (ii) drug mode of action; (iii) tumor specificities; and (iv) host reactions to the tumor and to treatment, in order to collectively deliver actionable information (Wargo et al., 2016). Often, these findings are non-obvious because they are based on contextual relationships of cells that represent key biology. They are nevertheless hugely relevant not only for stratifying efficacy and mode of action, but also for monitoring and predicting acute and chronic toxicities, modes of innate and acquired resistance, and for designing future meaningful clinical trials (Tang et al., 2018).

Certain anti-cancer drug-classes preceding CPIs, such as targeted biologics (trastuzumab, cetuximab) or even anti-hormonals (tamoxifen, aromatase inhibitors), to list a couple, landed themselves to a strategy of a single analyte biomarker (HER2 and estrogen receptor, respectively). This approach measures whether the drug target itself is present in the tumor of interest, which in turn drives therapeutic decision making and stratifying patients to relevant treatments.

However, this approach is suboptimal when it comes to immunotherapies in general. For example, patients that are diagnostically PDL-1 positive have widely varying responses to treatment, while treatment of PDL-1 negative patients with the same drugs can still achieve unexpected efficacy. Identifying mechanisms and biomarkers of both response and resistance to CPIs, ideally at an individual patient level, would be of significance for further tailoring the use of approved immunotherapies and for more rapid and successful development of novel immunotherapeutic approaches (Blank et al., 2016; Chen and Mellman, 2017).

**FIGURE 1:**
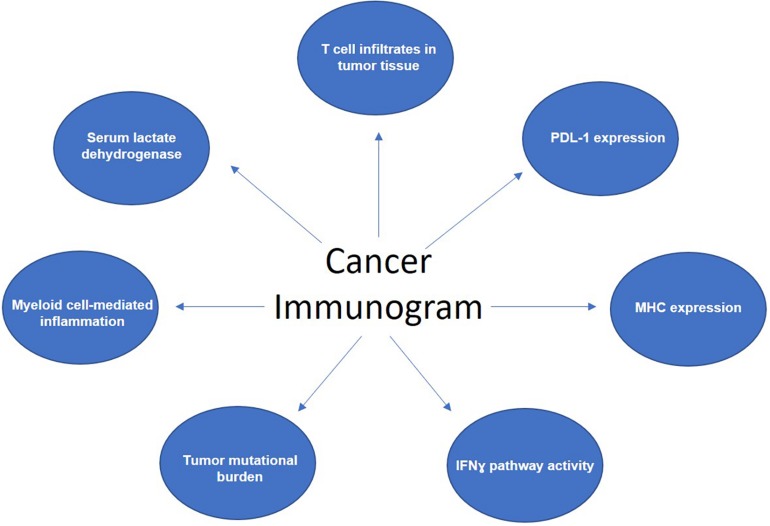
Components of the cancer “immunogram.”

Biomarkers available today could be broadly classified into three categories: (i) biomarkers that tell us if the tumor is “inflamed;” (ii) those that reveal the “immunogenicity” of the tumor – that is, how likely is it to engage an immune response; and lastly (iii) the biomarkers of the “host” factors. Seven parameters, which reflect and affect anti-cancer immune response form basis of the cancer “immunogram” framework and capture the three mentioned biomarker categories (Blank et al., 2016). These are: (i) degree of T cell infiltrates within tumor tissue; (ii) PDL-1 expression levels; (iii) MHC expression; (iv) IFNɣ pathway activity as a measurement of how sensitive cancer cells may be to effector T-cell killing; (v) tumor mutational burden; (vi) parameters of myeloid cell-mediated inflammation, such as the C-reactive protein (CRP) and IL-6 levels, and (vii) serum lactate dehyrogenase (LDH) levels, which can be indicative of the tumor burden (Figure 1). The immunogram, therefore, functions based on the premises that the involvement of active and proficient cytotoxic T cells is the ultimate effector mechanism in human tumors.

After several years of great efforts in the immunotherapy biomarker space, today, there are only two predictive biomarkers endorsed to guide decision-making about prescribing checkpoint inhibitors: expression of the programmed death-ligand 1 (PDL-1) and the microsatellite instability (MSI) status of the tumor.

## PDL-1 – Is This the Best We Have So Far?

Programmed death receptor 1 (PD-1), the target for approved anti-PD-1 inhibitors (nivolumab, pembrolizumab, and cemiplimab), is found on activated T cells and inhibits T cells from attacking tumor cells. Target molecules of approved CPIs, namely the programmed death receptor-1 and its ligand PDL-1 (approved anti-PDL-1 agents are atezolizumab, durvalumab, and avelumab), affect both tumor progression and related patient survival, by hindering tumor- neutralizing immune surveillance (Alsaab et al., 2017). If PDL-1 or PD-1 are blocked, then the immune system can be unleashed to attack cancer cells. PDL-1 expression on tumor cells and/or within the tumor microenvironment can indicate a pre-existing anti-cancer immune response. Despite considerable research and clinical efforts, we remain deprived of unequivocal data clarifying the precise link between PDL-1 expression levels on tumor cells or on tumor-infiltrating immune cells and clinical responses to CPIs (Herbst et al., 2014). What we do know is that the mechanism regulating PDL-1 expression in one and the other compartment may very well be distinct (Kim et al., 2016). How much PDL-1 expression is necessary for a clinically meaningful response to PD-1 or PDL-1 blockade is not well quantified and varies across different cancer types (from ≥1% to ≥50% positive PDL-1 cells), and even different checkpoint inhibitors. In some non-small cell lung cancer (NSCLC) studies, PDL-1 levels equal to or exceeding 50%, based on the so called proportional score, confer a favorable response to anti-PD1 blockade (Haslam and Prasad, 2019). In direct contrast, in another phase III trial (CheckMate-026), nivolumab activity in NSCLC was independent on PDL-1 levels, introducing the complexities around using PDL-1 as a sole predictive biomarker (Catenacci et al., 2019). An approach to combine ≥25% positive PDL-1 expression levels on tumor cells and on immune cells into a so called composite score has been used, for example in urothelial cancer and has proven indicative of response to durvalumab.

As such, it was instigated as a pre-requisite test in this setting by the FDA (Chow et al., 2016). The plot thickens, however, due to the fact that different scoring systems for PDL-1 can be used in the same tumor type, but are pertinent to different CPIs. For example, in bladder cancer patients who are ineligible to receive cisplatin-based regimens, PDL-1 score on immune cells covering >5% on immune cells is required for prescribing atezolizumab. For the use of pembrolizumab in the same setting, on the other hand, the recommendation is to utilize the combined score, which needs to be >10% and takes both PDL-1 on tumor and immune cells into account. It is no easier in lung cancer. Implementation of pembrolizumab first line calls for >50% of PDL-1 positive tumor cells, while in the second line metastatic NSCLC setting, that requirement is only a >1% score. In other cancer types as well, such as head and neck squamous cell, and Merkel carcinoma, PDL-1 positivity favors a response to PD-1 blockade (Chow et al., 2016; Massard et al., 2016). Nevertheless, in melanoma and bladder cancer, even patients with tumors that are negative or low for PDL-1 expression derive some level of clinical benefit from anti-PD-1/PDL-1 inhibitors. Meta-analyses of eight randomized controlled trials with avelumab, atezolizumab, durvalumab, nivolumab, and pembrolizumab, encompassing >4000 patients with advanced or metastatic cancers, confirmed that in both patients that were PDL-1 positive and PDL-1 negative, the long term clinical benefits from PD-1 or PDL-1 blockade could be observed. This introduces and argument for the predictive value of PDL-1 expression as a biomarker for outcome, and PDL-1 expression status, while informative and often reassuring, may indeed be insufficient in determining which patients will respond versus fail on PD-1 or PDL-1 blockade therapy (Shen and Zhao, 2018). This is even more so true in the new era of combining chemotherapy plus immunotherapy as frontline standard of care in multiple indications.

Programmed death receptor-1 immunohistochemistry (IHC) has been the primary diagnostic assay for assessing the expression of this marker in the clinic. Major testing and clinical decision making limitations still exist and are reflected in using different antibodies, platforms, scoring systems, tissue sampling limitations, focal and dynamic PDL-1 expression and different scoring cut-offs (Cesano and Warren, 2018). Blueprint Working Group has been established as an initiative to analyze and compare PDL-1 IHC assays, which are in routine use or under development. Two anti-PDL-1 antibodies, Ventana PDL-1 (SP142) assay and the PDL-1 IHC 22C3, are FDA approved. Two more are being viewed as complementary diagnostics PDL-1 IHC 28-8 and PDL-1 IHC (SP263). The 22C3 assay is indicated for NSCLC, gastric/gastro-esophageal junction adenocarcinoma, cervical cancer, and urothelial carcinoma, and treatment thereof with pembrolizumab. Two main PDL-1 scoring systems have been established: the tumor proportion score (TPS), and the combined positive score (). TPS finds utility in NSCLC for example, where the percent viable cancer cells that demonstarate complete or partial PDL-1 membrane signal are quantified. If TPS is ≥1%, the score may be considered positive, while to assign a high PDL-1 TPS, the score must equal or exceed 50%. Other cancers utilize the CPS, where the number of PDL-1 positive tumor cells and immune cells (lymphocytes and macrophages) gets divided by the total number of viable tumor cells, with the resulting number being multiplied by 100. Different PDL-1 CPS cut off values constitute positivity in different cancer types. For example, in urothelial carcinoma, CPS ≥10 renders the tumor PDL-1 positive, while in other carcinoma types, it is the CPS of ≥1. The PDL-1 (SP142) assay is indicated for urothelial carcinoma and NSCLC. In this assay tumor cells and tumor-infiltrating immune cells are assessed and scored separately. In urothelial carcinoma, it is sufficient to score tumor infiltrating lymphocytes only and the score of ≥5% confers eligibility for atezolizumab. For NSCLC, both the tumor and the immune cells need to be scored and PDL-1 expression of ≥50% on tumor cells or ≥10% on immune cells is linked to better survival outcomes on atezolizumab.

Programmed death receptor-1 epitopes detected by these antibodies are not necessarily the same. For example, clone 28-8 has been generated using a PDL-1 peptide extracellular domain and clones SP142 and SP263 using the cytoplasmic peptide. Consequently, the proportion of PDL1-stained tumor cells tends to be comparable when the 22C3, 28-8, und SP263 assays are used (Hirsch et al., 2017), while the clone SP142 is known to stain immune cells more intensively. Moreover, non-uniform, cross-comparative PDL-1 tissue IHC results may also result from epitope instability upon prolonged specimen fixation or inadequate tissue handling. Collectively, such challenges call for rigorous standardization of tissue processing and PDL-1 immunohistochemistry protocols.

Tissue heterogeneity gives high probability of obtaining false negative results or underestimating PDL-1 positive patients, simply because the tissue fragment undergoing analyses has been obtained from the tumor section which happens to be devoid of PDL-1, while not excluding the possibility that other parts of the same tumor mass contain PDL-1 expressing cells. Obtaining multiple core biopsies instead of just one, therefore ensures that as much of the intra-tumoral diversity is captured, and consequently interrogated for biomarker expression. Supporting this notion, studies have indeed verified that a multi-core approach yields better predictive results over a single core procedure (Munari et al., 2018). Considerations of how tissue storage over prolonged periods of time (months or years), and treatments administered post-tissue acquisition (radiation therapy, chemotherapy, or kinase inhibitors), may have affected PDL-1 expression at the time when the PDL-1 status from archival tissue samples is used to determine the course of treatment initiation, must be applied (Topalian et al., 2016). Collectively, this highlights the critical aspects spanning from tumor biology, treatment history, timing and method of tissue sampling and handling, to diagnostic antibody selection, processing and scoring, as determinants and caveats for successful implementation of PDL-1 as a clinical biomarker.

Several variations of measuring PDL-1 in biospecimens have been under consideration in order to complement or improve upon PDL-1 IHC assays. PDL-1 is a glycoprotein with heavy N-glycosylation residing in its extracellular domain, as a consequence of post-translational modifications, and assessing deglycosylated forms of PD- L1 in tissues has been proposed as a potential superior biomarker over current assays. When a study in 200 patients with various solid tumors (breast, lung, colon, prostate, and pancreatic cancers), interrogated PDL-1 levels before and after deglycosylation, it strikingly demonstarted a >2-fold increase in PDL1-positivity at 47.5% (by H-score) when deglycosylated PDL-1 was taken into account. The same result was subsequently validated in three separate cohorts of NSCLC, where the PDL-1 TPS score increased in 22.5% of patients from <1% to ≥5% and in 16% of patients from <1% to ≥49% following deglycosylation. Overall, in the NSCLC cohorts studies, PDL-1 status changed from negative to positive in ∼16% of patients (Sidaway, 2019). By this re-scoring, many more patients would have been eligible for treatment with nivolumab.

Interestingly, measuring PDL-1 levels using RNAseq may be promising and comparable to results obtained by IHC in terms of the predictive value for CPI response. A large cohort of over >400 patient tumors was analyzed by both techniques demonstrating these findings. For example, in melanoma patients, there was a strong association of objective response rate to high PDL-1 RNA-seq expression, even regardless of PDL-1 IHC results. Some advantages warrant further assessing and perhaps validating PDL-1 RNA-seq as an approach that offers superior standardization and less interpretation bias than IHC (Conroy et al., 2019). Obtaining serial tumor tissue biopsies is highly challenging, amount of tissue is often insufficient, and high bleeding risk often prevents serial tissue sampling. Another more available matrix for serial detection and measurement of PDL-1 is therefore patients’ serum/plasma. Both tumor and immune cells can be the source of the soluble, circulating PDL-1 protein. It can be detected using standard ELISA assays in patient blood, with high PDL-1 levels associating to adverse prognosis in multiple tumor types. In NSCLC for example, high soluble PDL-1 is associated with adverse prognosis, and studies in melanoma suggest it could even be utilized as a dynamic predictor of durable efficacy to checkpoint inhibitors (Hofman et al., 2019). Mechanistically, it is plausible that elevated soluble PDL-1 levels reflect an immunocompromised/suppressed state, and as such interfere with CPI efficacy (Zhou et al., 2017). However, even in this setting, we are faced with confounding observations because certain studies report that an increases in soluble PDL-1 upon checkpoint inhibitor treatment is associated with clinical responses. Therefore, soluble PDL-1 dynamics becomes an important parameter to consider when looking into this assay for further biomarker development (Zhou et al., 2017; Okuma et al., 2018).

Measuring PDL-1 expression on the surface of circulating exosomes has also been considered in the context of PDL-1 biomarker development. Exosomes, extracellular vesicles generated and released into the blood, are stable and abundant in circulation, and have been known to not only carry genomic cargo, but also to express biologically relevant surface proteins, such as PDL-1.

Exosomal PDL-1 is able to bind PD-1 expressed on immune cells, thus actively suppressing effector CD8^+^ T cells. Consequently, high levels of PDL-1 on exosoms preceding anti-PD-1 therapy, has been linked with worse prognosis in melanoma patients. Although exosomal isolation and detection parameters remain non-standardized, stability of these vesicles suggests that relying on exosomal PDL-1 might be more reliable compared to soluble PDL-1 levels (Chen G. et al., 2018). In head and neck cancer, for example, PDL-1 levels on exosomes, but not levels of soluble PDL-1, associate with disease progression (Theodoraki et al., 2018). In melanoma, exosomal PDL-1 was detected in all (100%) patients whereas of those, 67% had corresponding PDL-1 tumor positivity. Although baseline exosomal PDL-1 may not correlate with clinicopathologic characteristics and melanoma tumor burden, exosomal PDL-1variations before and after treatment seem to correlate with tumor response to CPIs and to survival. A differential of >100 was defined as a cut-off for PDL-1 exosomal levels between pre- and post-CPI treatment (bearing 83% sensitivity, 70% specificity, a 91% positive predictive value and a 54% negative predictive value), as meaningful in relation to tumor progression (Nardin et al., 2019). Another avenue for capturing PDL-1 levels is detecting it on circulating tumor cells (CTCs). Studies in NSCLC have pointed toward potential clinical significance of PDL-1- positive CTCs (Kloten et al., 2019). The endeavor is, to date, still in its infancy, and remains difficult to develop and optimize. CellSearch^®^ System, is the only FDA approved CTC methodology with some clinical utility in breast, colorectal and prostate cancers. It relies on capturing epithelial cell adhesion molecule (EpCAM) positive tumor cells from whole blood, however, its broad clinical value remains elusive. Determining PDL-1 expression on CTCs, in addition to all preceding challenges of this technology (namely false positive or negative assay results) and ability to ensure specificity, heightens the risk for co-isolating cellular populations like myeloid cells, and misidentifying them as CTCs expressing PDL-1 (Hofman et al., 2019).

In addition to PDL-1, its counterpart ligand, PDL-2, can also bind to the PD-1 receptor. However, PDL-2 has not gained the same level of attention and investigation as a biomarker of interest. Nevertheless, existing data do point toward the contribution of PDL-2 to immune response in that its high expression in head and neck cancer patients treated with pembrolizumab predicted worse response and shorter survival. Moreover, high PDL-2 mRNA expression in renal cell carcinoma, melanoma, metastatic urothelial and NSCLC tumors, correlated with PDL-1, and high PDL-1 and PDL-2 mRNA were both independently associated with improved outcome to atezolizumab (Yearley et al., 2017).

### Microsatellite Instability Status – Poster Child Biomarker for Tumor Type Agnostic Immunotherapy Approval

In 2017, FDA granted an umbrella approval for pembrolizumab for the treatment of patients with unresectable/metastatic, both adult and pediatric solid tumors harboring microsatellite instability high (MSI-H) or mismatch repair deficient (dMMR) tumors. This was a true landmark achievement and the first cancer treatment approved based on a common biomarker and not based on tumor type (Zhao et al., 2019). In determining the MSI/MMR status, four proteins which constitute the DNA mismatch repair pathway are assessed in tumor tissue. These are: (i) melanocyte-stimulating hormone 2 and 6 (MSH2, MSH6); (ii) MutL homolog 1 (MLH1); (iii) post-meiotic segregation increased 2 protein (PMS2); (iv) DNA polymerases (POLE). If one or more of these markers are not detected through immunohistochemistry, the status is called mismatch repair deficient (dMMR). On the other hand, the same proteins’ cognate genes can be inactivated due to germline (hyper)mutational changes, effectively resulting in what we refer to as high microsatellite instability (MSI-H). Common sites which are found mutated and hence are routinely tested in MSI panels are *BAT25* and*BAT26* (two nucleotide repeats) and *D5S346*, *D2S123*, and *D17S250* (three dinucleotide repeats). dMMR and MSI-H are typically highly concordant, allowing for the two terms to be used practically interchangeably in routine clinical practice (Cicek et al., 2011). Since this unprecedented decision by the FDA to award a “blanket” approval for the drug in dMMR/MSI-H cancers, the Agency has continued to prioritize an equivalent type of clinical trial design (Boyiadzis et al., 2018).

## Tumor Mutational Burden (TMB) – Leading Immunotherapy to the Era of Precision Medicine

It is well known that malignant tumors harbor somatic mutations which can be measured as, and reflected by the so called neoantigen load or tumor mutational burden (TMB). This feature of cancers has emerged as an attractive and relevant potential biomarkers of response to CPIs (Chan et al., 2019). Mutational burden is captured as a continuous variable, which raises the question of defining cut-off values to fairly reflect how different TMB levels affect response to immunotherapy (Allgauer et al., 2018). Whole exome sequencing (WES) is a desired approach for capturing and enumerating coding and somatic mutations in tumors, based on which TMB is computed. Assessing and comparing targeted next generation sequencing (NGS) panels to WES for assessment of TMB have yielded data which thus far appear to be in alignment (Buttner et al., 2019). Two providers established their respective, proprietary targeted NGS tests: (i) Foundation Medicine assays 324 genes to compute numbers of coding gene mutations (synonymous and non-synonymous). Further processing in this assay entails subtracting germline mutations using bioinformatics from the numbers of somatic mutations. The Foundation One assay deems the score >10 as TMB high, and this load predicts the response to various checkpoint inhibitors (Truesdell et al., 2018). The second NGS approach comes from the Memorial Sloan Kettering Cancer Center and is dubbed MSK-IMPACT. This is a 468 gene panel assaying tumor and matched blood samples in order to ultimately count non-synonymous mutations, from which germline mutations are then subtracted, to derive a TMB high value of 7 and higher (Zehir et al., 2017). The highest challenge in routine clinical practice where these targeted gene panels are used, is determining the clinical relevance, implications and optimal therapeutic course of action for patients with an intermediate TMB score. The FDA approved both assays in 2017 on the back of readouts from multiple clinical trials (Allegretti et al., 2018).

These approvals stem from multiple lines of evidence from clinical trials listed here: (i) in 2014, the first report of TMB effect on response to ICB in melanoma; (ii) in 2015, KEYNOTE-001 shows that TMB is associated with durable clinical benefit in 2L + NSCLC; (iii) in 2016, IMvigor210 demonstrates that response to atezolizumab is related to TMB in 2L + bladder; (iv) FIR/BIRCH/POPLAR: TMB assessment by Foundation One in 2L + NSCLC; (v) that same year, FIR/BIRCH/POPLAR looks at and shows TMB association with efficacy in 1L and 2L + NSCLC; (vi) In 2017, CheckMate -026 sees high TMB associated with response in 1L NSCLC; (vii) IMvigor210 trial demonstrates that TMB is associated with response in 1L bladder and (viii) KEYNOTE-012/KEYNOTE-028reveal how TMB is associated with best overall response in 1L + solid tumors; (ix) later in 2017, CheckMate-275 shows that high TMB associates with survival in 2L bladder and (x) CheckMate-032 that high TMB associates with survival in 1L + SCLC; (xi) also in 2017, OAK/POPLAR study analyzed TMB in 2L + NSCLC, while (xii) BFAST and B-F1RST trial validated TMB assay in 1L NSCLC; (xiii) toward the end of a successful 2017 year for the TMB assay, CheckMate-038 trial underscored how TMB associates with survival in ipilimumab naive patients in 2L + melanoma; (xiv) in 2018, CheckMate-227 showed that high TMB associates with survival in nivolumab plus ipilimumab treated patients in 1L NSCLC. Prospective clinical trials continue to investigate TMB as a biomarker for CPI treatment (Chan et al., 2019). Disease-specific TMB thresholds for effective prediction of response in various malignancies are still not well established. However, despite clinical trials yielding promising correlations between TMB and response rates, as well as progression free survival on nivolumab (CheckMate-016, CheckMate-227), Brystol-Myers Squibb recently withdrew their supplemental biologics license application in which they seeked frontline approval for the nivolumab (Opdivo) and ipilimumab (Yervoy) combination in advanced, high TMB NSCLC patients. This underscores the fact that more refinement of the role of TMB in relation to immunotherapy is needed. Data stemming from the NSCLC CheckMate-026 and similar trials, for example suggest that TMB and PD-L1 are likely complementary biomarkers, since patients with high TMB and PDL-1 ≥50% had the best response of 75%, while only 16% of patients with lower TMB and PDL-1 levels benefited from therapy. This clearly points toward the fact that TBM and PDL-1 may be complimentary biomarkers, reflecting activity at different steps of the immunity cycle and the need for potential biomarker integration.

## Intra-Tumoral T Cells: Presence and Function Matter

The proportion and functional properties of the intra-tumoral immune infiltrate are both dynamic characteristics. They evolve through the lifespan of the tumor and at any given point in time respond to and reflect upon tumoral, microenvironmental, systemic and therapeutic signals and impacts. Moreover, these features can be, and most often are different between primary and metastatic disease, while distinctions may exist even between individual tumor lesion in oligometastatic patients. In order to honor and capture these complexities, the International Immuno-Oncology Biomarker Working Group developed guidelines to standardize assessment and scoring protocols of TILs in solid tumors (Hendry et al., 2017). There are confounding reports attributing relevance both to pre-existing as well as TME re-populating TILs. Analyses of single-cell T cell receptor (TCR) sequences revealed that only few pre-existing, exhausted T-cell clones present in tumors prior to therapy, expand after therapy. Instead, the majority of significantly expanded clones were new clonotypes not present prior to treatment. The expansion of new clones, is referred to as clonal replacement (Yost et al., 2019). Two independent cohorts, considered as discovery and validation sets, assessed and correlated the proportion of so called partially exhausted (PD-1high/CTLA-4high) tumor-infiltrating CD8^+^ T cells with objective response and progression free survival to pembrolizumab and nivolumab in melanoma patients, and observed a striking result.

Namely, patients whose tumors had more than 20% PD-1hghi/CTLA-4high intra-tumoral cytotoxic T lymphocytes (CTLs) had superior response, which translated into longer progression free survival, than patients with ≤20% PD-1high/CTLA-4high CTLs (response rate: 85.7% versus 0% and 78.6% versus 0%; progression free survival: 31.6 versus 9.6 months and 15.9 versus 9.9 months, in the discovery and validation cohorts, respectively). Functionally, these CTLs were able to produce IFNγ but not TNFα and IL-2 (Daud et al., 2016). An independent group determined that anti-PD1 regimens support functionally activated T cells. Namely, they found that responding patients’ tumors favored a change in frequency of central memory over naive TILs pre-treatment, and the increase in CTLA-4 and PD-1 expression, and IFN-γ, IL-17A, Granzyme-B production post-treatment (Krieg et al., 2018). Interestingly, when peripheral blood T cells were measured before and after treatment, they were found consistently reduced in the peripheral blood of responders as compared to non-responders. This phenomenon may indicate that, in responders, T cells from peripheral blood, proficiently and actively migrate into the tumor sites. Location of TILs can also impactresponse, and cytotoxic T cells at tumor margins were also linked to response to anti-PD-1 agents (Tumeh et al., 2014). Without characterizing the location or phenotypic/functional profile of TILs, merely their abundance was also shown to bear relevance. In multiple tumor types, median rate of TIL infiltration was 7%, with no difference according to tumor type. Median TILs percentage was higher in patient with response to checkpoint inhibitors (17.5% vs. 5%). Response rate in patients with TILs ≥7% was 32%, versus 9% when TILs% was <7%.

Given that TILs are deemed important effectors and executors of cytotoxicity, hence enabling efficacy of immunotherapies, there was a need for a system which would classify tumors according to their immune based rather than only cancer-centric characteristics. Immunoscore introduced the notion of “hot” and “cold” tumors, corresponding to highly infiltrated (Immunoscore I4) and non-infiltrated (Immunoscore I0) tumors, respectively (Galon and Bruni, 2019). In between is the “altered phenotype,” further divided into “excluded” and “immunosuppressed” tumors (Galon et al., 2012; Kumpers et al., 2019).

Immunoscore^®^ has been developed in colorectal tumors, and implements an IHC-based assay to test intra- and peri-tumoral T cell infiltration, and in doing so assesses thehost immune response. The immunoscore itself is meant to reflect the density of two lymphocyte populations, cytotoxic (CD8^+^) and memory (CD45RO+) T cells, in the core and at the invasive margins, yielding a four-point scoring scale which ranges from immunoscore 0 (I0; low densities of both cell types in both regions), to immunoscore 4 (I4; high densities of both cell types in both regions) (Mlecnik et al., 2016; Sun et al., 2019). Tissue availability is therefore of essence for performing the immunoscore test, and in itself represents a challenge, as was discussed in the context of PDL-1 IHC. Nevertheless, given the association between the presence of CD8^+^ and expression of PD-1 and PDL-1 in the tumor, immunoscore is an attractive potential predictive marker of response to checkpoint inhibitors, and certainly one worth further investigating across tumor types (Galon et al., 2006). In patients with NSCLC treated with durvalumab, improved survival was observed especially in CD8^+^/PD-L1^+^ tumors (24.3 months) compared with CD8^+^ (17.8 months) or PD-L1^+^ (17.1 months) (Shien et al., 2016). Although preliminary, these results are encouraging that an improved predictive composite TIL/PDL-1 biomarker system should be further evaluated (De Guillebon et al., 2020).

In order for the immune response to be triggered and then mobilized, the mare presence of T cells is insufficient, but the T cell receptor repertoire needs to be broad as well. From studies in melanoma patients treated with nivolumab and ipilimumab, and bladder cancer patients treated with atezolizumab, where T cell repertoire was measured in peripheral blood, we learnt that if the T-cell receptor repertoire is restricted or uneven, it can have a negative impact on survival (van Rooij et al., 2013*;* Snyder et al., 2017). In patients responding to anti-CTLA-4 therapy, no specific clones were expanded preferentially, inferring that a number of important clones get disinhibited and are allowed to proliferate. Some of these “disinhibited,” proliferating clones can also generate a proinflammatory or autoimmune hyper-responsiveness associated with immune related side-effects. Interestingly, in patients treated with anti-PD1, a more “focused” TCR repertoire is associated with response (Tumeh et al., 2014*;* Hogan et al., 2019). A commercial immune-sequencing assay, ImmunoSeq (Adaptive Biotechnologies), aims to ascertain both the specific, individual clones, as well as the complete CDR3 repertoire. It is therefore aimed at capturing the broad clonality of T cells, in order to infer the degree of expansion of tumor-reactive clones. The clinical utility of this particular approach has not yet been established.

### Tumor Neoantigens: The More the Merrier

Somatic mutations which are characteristic for malignant cells, give rise to so called neoantigens, enabling immune system cancer recognition as “non-self” and subsequent immune-mediated attack (Castle et al., 2019). Tumors with a high clonal neoantigen burden typically exhibit a favorable response to CPIs (Furness et al., 2016; McGranahan et al., 2016). A seminal paper in 2014 revealed that mutational load increases the probability of response to CPIs (*p* = 0.01), but that as a stand-alone, it is not sufficient to predict favorable outcome. The search for a neoepitope signature which may be associated with therapeutic benefit, revealed that patients who derived long-term clinical benefit on CPIs share a tetrapeptide neoepitope sequences, which is absent in non-responders or short-term responders. Interestingly, this tetrapeptide sequence is homologous to viral and bacterial antigens. implying that meaningful tumor neoepitopes appear to resemble those from pathogens, which T cells are likely to recognize and mount an immune response attack toward (Snyder et al., 2014). A technology named MANAFEST (Mutation-Associated Neoantigen Functional Expansion of Specific T Cells), incorporates multiple techniques including WES, T cell receptor sequencing and comprehensive bioinformatics, proposes actionable tumor mutations and even includes peptide-stimulated cultures, to ultimately achieve an all-encompassing, anti-tumor immunity surveillance. This approach is uniquely poised to gauge a patient’s own immune response to their tumor, but whether its clinical utility will be superior in patients with high or low neoantigen burden, remains to be determined (Danilova et al., 2018). Different mutation loads in tumors are also present in virus-associated cancers, characterized by a specific immunological profile (Sivanandam et al., 2019). Carcinogenic viruses such as the human papilloma virus (HPV), herpes simplex B (HBV), and HCV, and Epstein-Barr Virus (EBV), contribute toward development of ∼20% of human malignancies, and these tumors may respond better to CPIs (Varn et al., 2018). The rationale behind augmented efficacy of the PD-1 axis inhibitors in HPV + tumors may be attributed to higher PDL-1 expression, increased content of CD8 + T-cell infiltration, greater diversity of T-cell receptors and higher TMB (Wang et al., 2019). Pooled analysis of PD-1/PDL-1 inhibitors’ efficacy in a total of five hundred and eighty nine HPV-positive and -negative head and neck squamous cell carcinoma patients, from six trials (CheckMate-141, KEYNOTE-012, KEYNOTE-012 Expansion 11, KEYNOTE-055, NCT01693562, and NCT0137584212), showed a 21.9% vs. 14.1% response rate in favor of HPV + tumors. HPV turned out to be a predictive biomarker, independently of PDL-1 expression, and correlated with increased cytotoxicity and a T cell-inflamed microenvironment. Patients who had MSI-H or EBV-positive tumors (usually mutually exclusive), had dramatic responses to pembrolizumab, i.e., 100% response in EBV-positive metastatic gastric cancer, for example (Kim et al., 2018). In EBV associated non-Hodgkin (NHL) lymphoma, PDL-1 expression was significantly higher (56%) than in EBV-negative NHL (11%), further confirming that presence of viral oncoproteins is capable of rendering tumors an effective target for checkpoint inhibitors.

## Prediction Gene Signatures: Signal That Needs to Come From the Right Place

Nature Medicine Journal not too long ago featured a back to back coverage of two gene signatures named IMPRES and TIDE, as potential valuable strategies for predicting CPI treatment success. Immuno-predictive score (IMPRES), was validated on a total of 297 patient samples stemming from ten datasets of patients treated with anti-PD-1 and anti-CTLA-4. IMPRES was derived from a hypothesis that the same components governing spontaneous tumor regression in cancer, in this case neuroblastoma, may be the major determinants of immune responses to checkpoint inhibitors. With this postulate, 108 neuroblastoma patients with spontaneous regression and progressive high risk were analyzed and a gene signature score emanated that stratified individuals with a proficient immune response. This score (0–15) takes into account 15 pairwise correlations between the expression levels of inhibitory and activating immune-checkpoint genes, and higher scores predict higher likelihood of spontaneous regression. Immune stimulatory molecule genes (HVEM, CD27, and CD40) were associated with a more favorable response to anti-PD-1 and anti-CTLA-4. High expression of immune inhibitory molecules (CD276, TIM-3, CD200, and VISTA) was, conversely, associated with a worse response (Auslander et al., 2018). A contemporaneously identified gene signature, the tumor immune dysfunction and exclusion (TIDE) framework, used data from >33,000 human tumors and tested the effects that interactions between candidate cytotoxic T cell or immunosuppressive T cell genes had on response patterns to CPIs (survival or risk of death). Having been developed as such, TIDE provides signatures of T cell function in immunologically “hot” tumors and T cell exclusion in “cold” tumors. TIDE signature was pressure tested on pre-CPI treatment tumor tissue samples of melanoma patients and in this cohort it performed better in predicting response patterns than PDL-1 levels, TMB, and an IFNγ signature (Jiang et al., 2018). TIDE and IMPRES will undoubtedly be tested next in other cancer types with distinct immunological phenotypes, despite more recent dialogue which has put the statistical power and generalizability of IMPRES into the spotlight (Auslander et al., 2019; Carter et al., 2019). A smaller, prospective study conducted in NSCLC patients, collected pre-anti-PD1 treatment samples to analyze a 395 gene, immune-related signature (382 functional genes associated with lymphocyte regulation and markers, cytokine signaling, checkpoint molecules; 2 negative control genes and 11 housekeeping genes) (Hwang et al., 2020). Four result components were significantly correlated to durable versus non-durable responses. Those were the M1 macrophage and T cells signatures from the peripheral blood mononucleocytes, as well as high expression of PSMB9 and CD137 genes. These four components collectively, outperformed PDL-1 IHC, TMB or the TIL score in predicting survival benefit. While promising, the results will need to be confirmed in a much larger study.

Sequencing results can often be blurred due to the fact that very few tumors undergo laser capture microdissection of cancer cells, hence the diagnostic signal source cannot be attributed solely to the tumor vs the microenvironmental cellular content. To address this challenge, ESTIMATE (Yoshihara et al., 2013) and CIBERSORT (Chen B. et al., 2018) as novel methodologies using computational approaches tend to distinguish gene expression signatures and assign them to the distinct fractions of tumor cells, immune or stromal cells. Another transcriptomics based assessment is the 18-gene, tumor inflammation signature (TIS: TIGIT, CD27, CD8A, PD-L2, LAG3, PDL-1, CXCR6, CMKLR1, NKG7, CCL5, PSMB10, IDO1, CXCL9, HLA.DQA1, CD276, STAT1, HLA.DRB1, and HLA), which uses NanoString nCounter to measure intra-tumoral adaptive immune responses. It has originally been developed by Merck as a clinical grade trial assay, aimed at predicting responses to pembrolizumab (Ayers et al., 2017). The tumor inflammation signature consists of genes, which in an IFN-γ-dependent fashion, impact antigen presentation, expression of chemokine mediators, cytotoxic activity, and adaptive immunosuppression. NanoString have established a comprehensive, 770-gene panel called PanCancer IO 360^TM^, which interrogates individual tumor compartments as well, namely the tumor itself, the stroma and the immune infiltrate. Selected genes reflect not only the immune state (degree of activation versus immune evasion), but also confer mechanistic information about pathways present in the tumor, which may overall be impacted through therapeutic intervention. The IO 360 panel is still positioned for research rather than diagnostic purposes only, until further validation (Chen L. et al., 2018; Garris et al., 2018; Li et al., 2019). In the endeavor to interrogate the genomic landscape which may favor immunotherapy response over evading immune surveillance, the immune signature (IS) 105 gene panel emerged, and turned out to be significantly associated with response to immunotherapy. Thus far, the IS has been studied in melanoma patients treated with ipilimumab. The IS was compared to gene expression data from 30 different tumor type TCGA datasets, encompassing over 9000 patients. What transpired was that malignant tumors of the lymphoproliferative tissues, have a very high IS score, which served as validation, to some extent, that the signature itself is well suited to reflect immune activation patterns in analyzed tissues. Two immune cell types were most significantly driving the IS, namely the fraction of CD8^+^ T cells and the immune-proficient macrophages (M1). The ultimate analyses breaks down the tumor assignment into two classes: the C-type tumors likely to be resistant to immunotherapy, and the M type tumors likely to respond on the count of somatic mutation enrichment (Ock et al., 2017). An additional pan-tumor T cell-inflamed gene expression profile (GEP) was assessed in 220 patients with 9 cancers. RNA was acquired at baseline from patients that went on to receive pembrolizumab and IFN-γ-responsive genes related to antigen presentation, chemokine expression, cytotoxic activity, and adaptive immune resistance appeared highly expressed in ultimate responders (Ayers et al., 2017). The same signature was further ran against twenty-two cancer type and an >300 patient collection from four other pembrolizumab (KEYNOTE) studies, where its independent predictive value of response (*p* = 0.005) was confirmed (Cristescu et al., 2018). While the features of the GEP seem to be necessary in responders, they are not always sufficient for clinical benefit, and this reiterates the complexity behind actionable clinical implementation of any gene signature profile on its own.

In addition to the gene signatures that once they are derived from the suitable biospecimen, provide a snapshot of the intra-tumoral and patient’s immune-biology and immune-mechanics, certain tumor cell specific oncogenic/onco-suppressive genetic events may also play a role in determining and directing immune response. Patients with metastatic, KRAS-mutated lung cancer, treated with at least one cycle of a PD-1 inhibitor or an anti-PD-1/PDL-1 and anti-CTLA-4 combination regimen, had STK11/LKB1 mutations largely drive the primary resistance to PD-1 blockade, in any regimen (Skoulidis et al., 2018). Interestingly, in patients with combined TP53 and STK11 alterations, response was far superior (57% response rate) than in KRAS-STK11 mutation carriers (0% response rate), who were virtually non-responders, in the CheckMate-057 trial (Mhanna et al., 2019). Besides the newly acknowledged relevance of STK11 in lung cancer, this disease is characterized by other, well known genetic abnormalities, such as EGFR, BRAF^*v*600*E*^ mutations, ALK, ROS, or RET rearrangements, as well as MET^*exon*^ ^14^ mutations. EGFR mutant tumors have worse response to overall CPIs, though outcomes can vary by allele.

Overall, experience has demonstrated that patients with tumors harboring an exon 19 deletion have lower response rates to CPIs than patients with tumors that are EGFR wild type or have the EGFR^*L*858*R*^ mutation (Hastings et al., 2019). In melanoma, the most prevalent oncogenic mutations are of course those in the BRAF gene. In patients with BRAF^*V*600^-mutated melanoma, BRAF and MEK inhibitors yield very high initial responses. Interestingly, as opposed to some EGFR mutant lung cancer, CPIs seem to work fairly well in BRAF-mutant melanoma. In particular, a doublet of an anti-PD-1 and an anti-CTLA-4 delivers superior benefit over a single agent approach in the BRAF-mutant setting. Nowadays, even triple combinations are being studied, and that of BRAF plus MEK inhibitors with PD-1 pathway blockers appears promising (Ascierto and Atkins, 2019). Globally, in patients with gene mutations conferring oncogene addiction, response rates to single agent immunotherapy are impaired compared with wild-type patients, and combination strategies should be considered and implemented.

### Epigenetic Modifiers in Immunotherapy

Epigenetic markers switch genes “on” and “off” in response to extracellular signals. These modifications converge on complex cellular processes that can alter cellular functions without affecting the actual genetic code. Epigenetic studies in lung cancer have shown that promoter regions of the CTLA-4, PD-1, and PDL-1 genes can be hypomethylated, which in turn associates with upregulated expression of these genes in the tumor microenvironment (Marwitz et al., 2017). Additionally, circulating microRNA signatures associated with survival on nivolumab, have been studied as well (Halvorsen et al., 2018). MiRNA-34a is can suppress PDL-1 expression in colorectal cancer and NSCLC, thereby inducing CD8^+^ TILs (Li et al., 2016), so the miRNA/PDL-1 axis could be considered as a potential therapeutic and/or diagnostic biomarker. It has therefore been postulated that epigenetic immunomodulation could prime the immune system for immunotherapy, and drugs targeting epigenetic machinery have been combined with checkpoint therapy (Dunn and Rao, 2017). DNA methylation has been studied in 18 cancer types in order to establish a relevant profile which could be correlated with the response rates to CPIs. This profile covers 191 genes from which 269 CpG signatures pertain to the developed profile (Xue et al., 2019). The CpG model out-performed the TMB score in many cancer types: adrenocortical-, bladder-, breast-, cervical-, endocervical-, esophageal, endometrial cancer, GBM, head and neck squamous cell carcinoma, kidney-, liver- lung cancer, mesothelioma, ovarian-, pancreatic cancer, melanoma and uveal melanoma (Xue et al., 2019). This type of an epigenetic assessment could be applied in conjunction with other immuno oncology biomarkers (such as TMB), to enable achieving better prediction performance in assisting oncologists’ selection of patients with higher likelihood of benefiting from PD-1/PD-L1 inhibition therapy.

### Liquid Biopsies: Do We Have a Surrogate for Tumor Sampling?

The importance and necessity to obtain fresh tissue samples for biomarker discovery are just as much appreciated as the challenges associated with standardized workflows to enable achieving this successfully. In therefore comes as no surprise that liquid biopsy (LB) is an approach, which is implemented in the context of targeted therapies, is now increasingly being studied in the immuno-oncology arena as an avenue for integrated biomarker analyses (Normanno et al., 2017). Many diverse analytes can be captured and measured from a liquid biopsy: circulating tumor cells (CTCs), circulating tumor DNA (ctDNA), proteins and cytokines, circulating T-lymphocytes and other immune cell types. Instead of assessing tumor mutational load and microsatellite instability from tissue specimens, researchers have been investing efforts in optimizing ways of profiling these tumor-associated characteristics from circulation (Khagi et al., 2017). For example, ctDNA concentration measured using large-cancer sequencing panels (NGS) pre-treatment and 2 months post-initiation of treatment, yielded correlations with clinical responses and survival benefit of nivolumab in advanced NSCLC. From a 22 gene panel, mutations in nine genes (TP53, NOTCH1, FBXW7, KRAS, SMAD4, KDR, DDR2, BRAF, and PTEN) were followed and found particularly useful in this context (Giroux Leprieur et al., 2018). Even pseudo-progression could be differentiated from true disease progression using ctDNA profiles, with sensitivity of 90% and specificity of 100% (Lee et al., 2018). So far, it appears that measuring ctDNA could enable earlier identification of patients who are likely to derive clinical benefit on anti-PD-1 antibodies, but it still needs to be determined that actioning treatment-related decisions purely based on ctDNA profiling early in the course of therapy, would not turn out to be ultimately deleterious for patients.

Serum protein and cellular markers are readily assessable, hence serial sampling throughout the disease and treatment course is routinely used in the clinic (Nishino et al., 2017). Multiple serum-based parameters have been investigated in response to pembrolizumab, as well as ipilimumab: lactate dehydrogenase (LDH), C-reactive protein (CRP), vascular endothelial growth factor (VEGF), as well as circulating CD25. Increased serum LDH before initiation of CPI therapy correlated with unresponsiveness to CTLA-4 blockade, and to a lesser extend also to anti-PD-1 treatment. Interestingly capturing a dynamic decrease between baseline and post-treatment initiation week 12, correlated with favorable response (Kelderman et al., 2014; Weide et al., 2016; Oya et al., 2017; Buder-Bakhaya and Hassel, 2018). CRP levels within normal range at baseline, and declining serum CRP between baseline and week 12 associated with better survival on ipilimumab in melanoma patients (Krieg et al., 2018; Galon and Bruni, 2019). For anti-PD-1 treatment in NSCLC, elevated baseline CRP levels associated with shorter progression free survival. Peripheral blood cell types are also important, and high neutrophil to lymphocyte ratio (NLR) ≥5 in NSCLC patients associated with poor progression free survival and overall survival treated with nivolumab and pembrolizumab (Russo et al., 2019). B cells are typically considered central in the adaptive immune system, and have only recently been linked to response to CPIs. Presence of a distinct B cell functional subset, called memory B cells, which can be detected in circulation and as tissue resident, associates with immune activation permitting checkpoint inhibitor efficacy. Memory B cell like profiles (MBL) and links to CPI response have been studied in urothelial carcinoma patients on anti-PDL-1 (*n* = 25), melanoma patients on anti-PD-1 (*n* = 28), and anti-CTLA-4 therapies (*n* = 42). Cell specific genes (TNFRSF17, MS4A1, and ADAM28), T cell function genes (CXCL9, CCL19, and CXCR3) and lastly genes reflecting MHC II antigen presentation (HLA-DQB1, HLA-DMA, and HLA-DPA1) comprise the MBL signature. This signature was associated with response to CPIs, regardless of the correlations obtained using other response predictors, such as TMB (Oya et al., 2017). B cells, therefore, may be particularly useful because one can capture changes in their frequency in peripheral blood, i.e., via a liquid biopsy, and this may link not only to response but also to an enhanced risk of immune related toxicities to CPIs. More specifically, PD-1/PDL-1 receptors can be expressed on the so called immunosuppressive B cells which can impede T cell dependent responses to checkpoint inhibitors and mediate ensuing immune adverse events. Specifically, reduced B cells count after CPI treatment, and an increase in PD1^+^ memory B cells, favor the development of immune toxicities. Quite uniquely, B cell count variations seem to be an early onset feature in this context, while changes in other peripheral immune cell types may occur only at later time-points (Liudahl and Coussens, 2018). Further involvement of T cell independent pathways in influencing response to CPIs, warrants discussion around myeloid-derived suppressor cells (MDSC) contributing toward, if not driving immunosuppression in various cancer types (Weber et al., 2018).

For example, melanoma patients resistant to ipilimumab tend to have high MDSC levels (Meyer et al., 2014; Martens et al., 2016). In addition, in NSCLC, when evolution of peripheral cell types was followed at baseline, and after 2 and 4 weeks post-therapy initiation, an accumulation of MDCSs was noticed in resistant patients. Those MDCSs expressed high levels of the immunosuppressive molecule galectin-9 (Limagne et al., 2019). This not only proposes a distinct cell type but also a unique mechanism associated with and implicated in primary and acquired resistance to check point blockade. Immune-suppressive cells’ ability to undermine the antitumor immune response has been observed in many tumor types, resulting in resistance to immunotherapy. Regulatory T cell (Treg) expression changes in patients on immunotherapy has particularly been interrogated in this context. The most confounding issue has been validating Treg biomarkers and distinguishing between different Treg subtypes both in peripheral blood and tumor microenvironment. So far, there is no consensus as to which Treg subset should be monitored and some focus has been placed on correlating CD4 + CD39 + CD25 + Treg cells with clinical response (George et al., 2019). A specific T cell subtype called immunosuppressive γδ T cells, have recently been implicated as culprits impacting response to ipilimumab. Immunosuppressive γδ1 T cells are observed in high percentages in blood and among TILs in many solid tumor types (7.2–75.7%; mean 33.2%), and are involved in inflammation-induced cancer progression. More specifically, melanoma patients with higher percentage of Vδ1 + cells in peripheral blood (≥30%) had poorer survival and a lower rate of clinical benefit on ipilimumab (Peng et al., 2007; Fleming et al., 2017). Monocytes and difference in their cell count before and during (at 12 weeks) of anti-PD1 therapy, have transpired as potential strong predictors of response and survival to anti-PD1 agents (Goswami et al., 2018; Krieg et al., 2018). Classical monocytes (CD14 + CD16-) are important for an effective anti-tumor immune response during anti-PD-1 immunotherapy. Increased expression of PDL-1 on monocytes correlated with not only a favorable initial but also a prolonged immune response and elevated levels of IFN-γ, which in a positive feedback loop, directly increases PDL-1 expression. Eosinophils have been considered the least likely cell type to associate with CPIs, but once studies took off, their relevance has been established, though it is insufficiently elucidated to date. In one of the larger studied melanoma cohorts (*n* = 173) for example, high eosinophil count (>20%) upon exposure to CPIs, favored clinical response (Moreira et al., 2017).

## Microbiome and PET Imaging – Do the Gut or Visuals Hold the Answer?

The relationship between the microbiome and the immune system is significant in human health and disease. There is an increasing understanding that microbiome-immune system interplay is a major factor influencing and driving immune phenotypes, and that gut flora composition can stimulate or inhibit immune responses. In alignment with this, an imbalance in the gut microbiota and low levels of a bacteria called *Akkermansia muciniphila*, are associated with impaired immune cell activity in patients not responding to checkpoint blockade. On the other hand, cartain microbes such as *Bifidobacterium longum*, *Collinsella aerofaciens*, and *Enterococcus faecium*, dominate in responders (Matson et al., 2018). Use of antibiotics before or shortly after checkpoint inhibitor therapy has been linked to worse response and survival in patients with renal and lung cancer (Routy et al., 2018; Otoshi et al., 2019). This accumulating knowledge has resulted in the development of multiple gut microbiome modulating therapeutics, which are now being extensively studied in combination with CPIs, in the clinical trial setting.

Novel imaging probes/biomarkers are also being integrated into standard techniques, e.g., for positron emission tomography (PET), and magnetic resonance imaging, in order to try and predict patients’ response, as early as possible. T cells specific imaging probes are able to, non-invasively, monitor systemic and intra-tumoral immune alterations during and after treatment with immuno-therapies (Wei et al., 2018). Tracers for PD-1 and PDL-1 have thus been developed (64CuNOTA-PD-1, 64Cu-NOTA-PDL-1, 89Zr-Df-nivolumab, 89Zr-pembrolizumab, 64Cu-pembrolizumab), and used in pre-clinical models and in some clinical trials. Results show that whole body PET imaging in patients with advanced NSCLC using these novel probes, detects PD(L)-1 expression, which correlates with results obtained using tissue IHC. Therefore, PET probes could have great clinical utility for longitudinal and non-invasive quantification of PD-(L)1 expression in the future of immunotherapy trials and routine practice (Niemeijer et al., 2018; Wei et al., 2018).

## Conclusion

**FIGURE 2:**
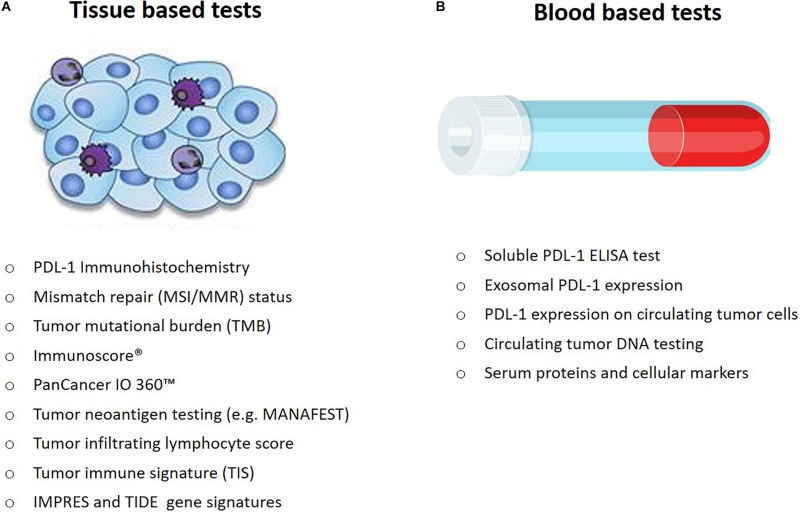
Approved and investigational prognostic and predictive markers that can be **(A)** measured from tumor tissue and **(B)** measured from blood, in cancer patients.

Armed with an ever growing armamentarium of candidate and some validated biomarkers, as well as with an increasing understanding of the complexities dictating response to CPIs, we can now state, with unequivocal conviction, that one-biomarker-fits-all, is not our reality in the immunotherapy landscape. A single biomarker accurately identifying patients across tumor types who will likely benefit from immunotherapy, is the holy grail, but one that is not within reach (Havel et al., 2019). The activity of immunotherapy is dependent on interactions between a person’s immune system and the tumor, both of which impact the outcome to any therapeutic intervention. Therefore, integration of the local, tumor and peripheral immune monitoring utilizing multiple techniques and methods, is necessary at multiple time points throughout immunotherapy treatment, to obtain a comprehensive systemic, organ, tissue, cellular and molecular immune mapping (Butterfield et al., 2018). Many of the predictive markers currently explored in laboratories worldwide will require extensive validation in prospective clinical trials (Figure 2). Oncology indications for approved checkpoint inhibitors will undoubtedly continue to multiply, in parallel with unprecedented drug development efforts to bring novel immunotherapies to life. These collective endeavors call for rapid incorporation of predictive therapeutic biomarker development and validation efforts, as an integral and indispensable component. Recently, the Foundation for the National Institutes of Health established the Partnership for Accelerating Cancer Therapies, as a long term commitment and effort to validate and standardize multifaceted biomarker assays to cater to the multitude of tumor types, which stand to benefit from cancer immunotherapies (Anonymous, 2018). The initiative aims to accomplish an admirable task: harmonization of assays that capture complex tumor-immune system interactions, tumor-intrinsic, immune microenvironmental, and host-related factors associated with drug response. An overarching theme in the immuno-oncology biomarker setting is the paramount importance of a multidisciplinary approach, utilizing the input from oncology specialists, pathologists, expert immunologists, and geneticists, tumor biologists, as well as bioinformaticians and statisticians. The desired common goal for drug developers, clinicians and patients is to effectively, precisely and personally tailor immunotherapy treatment regimens, using a multicomponent predictive biomarker system, which needs to be accessible, reproducible, sensitive, specific and cost-effective.

## Author Contributions

All authors listed have made a substantial, direct and intellectual contribution to the work, and approved it for publication.

## Conflict of Interest

AF and JB were employed by company PureTech *Health* PLC. The remaining author declares that the research was conducted in the absence of any commercial or financial relationships that could be construed as a potential conflict of interest.
